# Motor‐Assisted Co‐Migration of Intracellular Organelles and Microtubules as a Mechanism for Directed Cargo Transport

**DOI:** 10.1002/bies.70127

**Published:** 2026-03-25

**Authors:** Chuying Zhou, Mineko Kengaku

**Affiliations:** ^1^ Graduate School of Biostudies Kyoto University Kyoto Japan; ^2^ Shenzhen Key Laboratory of Neuropsychiatric Modulation Shenzhen‐Hong Kong Institute of Brain Science Shenzhen Institutes of Advanced Technology Chinese Academy of Sciences Shenzhen China; ^3^ CAS Key Laboratory of Brain Connectome and Manipulation, the Brain Cognition and Brain Disease Institute, Shenzhen Institutes of Advanced Technology Chinese Academy of Sciences Shenzhen China; ^4^ Guangdong Provincial Key Laboratory of Brain Connectome and Behavior the Brain Cognition and Brain Disease Institute Shenzhen Institutes of Advanced Technology Chinese Academy of Sciences Shenzhen China; ^5^ Institute for Integrated Cell‐Material Science (WPI‐iCeMS) Kyoto University Kyoto Japan

## Abstract

Intracellular cargo transport relies on a microtubule (MT) network and its molecular motors, dynein and kinesin. While conventional models emphasize motor‐driven cargo movement along stationary MT tracks, emerging evidence suggests that dynamic movements of MTs also contribute to directional transport. We propose a model of cargo co‐migration with moving MTs, exemplified by nuclear migration in developing neurons. This transport mode may operate across cell types, provided that cargo‐MT tethering and directional MT movements are present. We hypothesize multiple complementary mechanisms, including motor catch‐bond formation and clustering, as well as MT‐associated protein‐mediated anchorage. We further discuss how directional MT movements can be generated through motor‐driven sliding, cortical gliding, actin‐MT crosslinking, and dynamic MT instability. This coupled transport mechanism provides an additional layer of directional control that supplements motor‐stepping‐dependent transport. Potential experimental approaches to validate this hypothesis are discussed. Understanding MT‐mediated cargo delivery could refine our current models of intracellular transport and reveal new insights into neurodevelopmental and neurodegenerative disorders.

## Graphical abstract

Directional intracellular cargo transport is traditionally attributed to motor stepping along stationary microtubule (MT) tracks. We propose a complementary mechanism of cargo co‐migration with moving MTs. This mode requires cargo–MT tethering and directed MT motion. Future investigation will offer new insights into neurodevelopmental and neurodegenerative disorders.

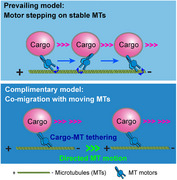

## 1 Introduction

**FIGURE 1:**
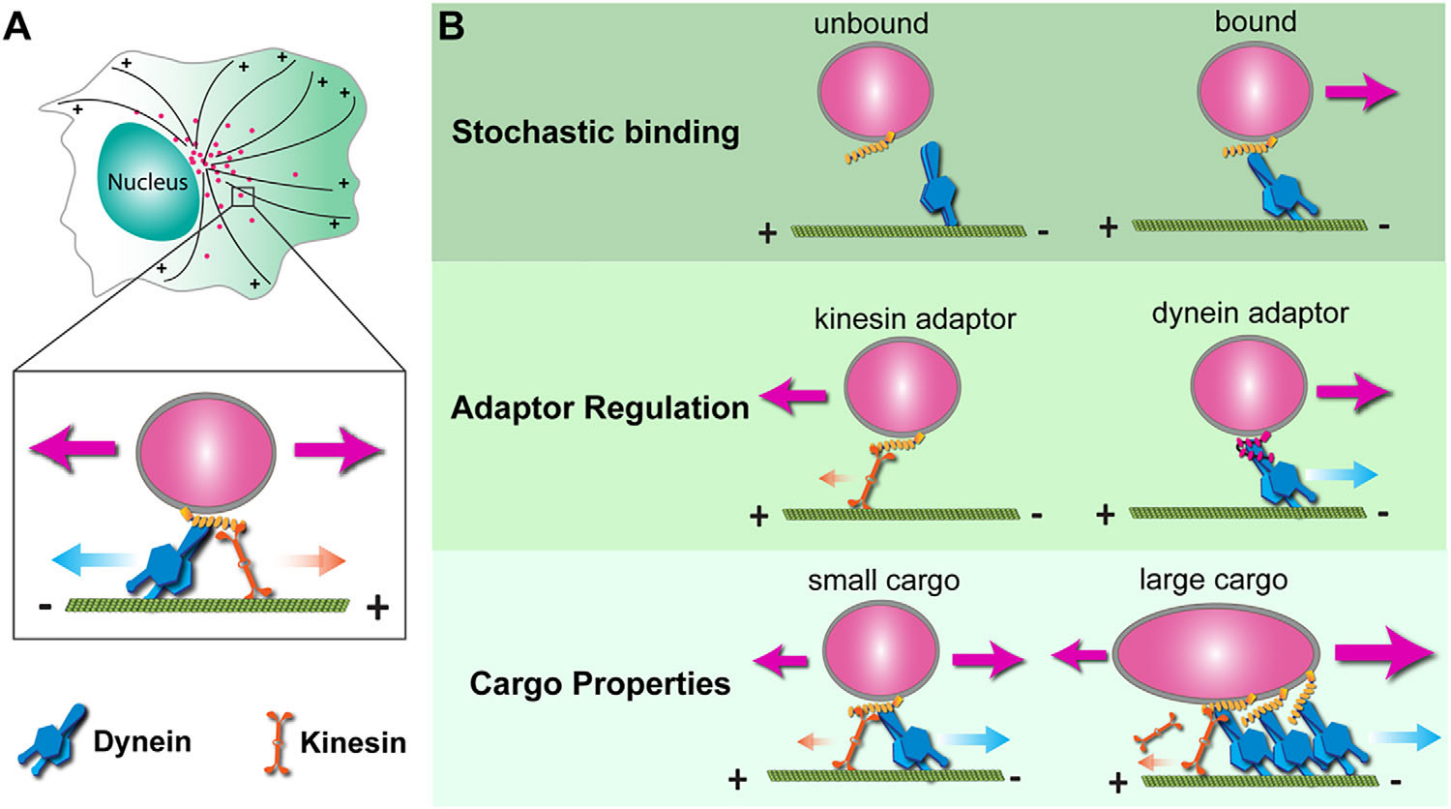
*Intracellular cargo transport by MT motors*. (A) In cells, transport of vesicles and organelles is driven by minus‐end‐directed dynein motors and plus‐end‐directed kinesin motors. (B) Directional cargo transport is determined by stochastic binding, adaptor regulation, and cargo properties. In the *Cargo Properties* section, increasing cargo size enhances the recruitment of additional motors, thereby promoting cooperative interactions among dynein motors. In contrast, kinesin motors, which lack the ability to cooperate in a similar manner, are unable to achieve efficient transport under these conditions.

In eukaryotic cells, long‐range transport of intracellular cargoes, including organelles, vesicles, and mRNA, relies on microtubule (MT) tracks. The molecular motors dynein and kinesin drive bidirectional transport along MTs, moving cargo towards the minus and plus ends, respectively (Figure 1A). Molecular and biochemical studies of motor complex assembly, activation, and inactivation, as well as the regulation of motor‐cargo‐linking adaptor molecules, have shed light on the fundamental principles governing intracellular transport [1, 2, 3, 4]. At the same time, biophysical analyses using advanced time‐lapse imaging of in vitro reconstitution of MT‐based transport and motor force measurement by optical tweezers have examined key parameters of motor‐driven transport, including speed, dissociation rates, and stall forces [5, 6, 7, 8]. However, a considerable gap remains between in vitro findings and in vivo observations due to the complex intracellular environment in living cells. Here, we propose a complementary mechanism influencing MT‐based cargo transport: rather than relying solely on motor stepping activities, cargo movements also synergize with the dynamic movements of MT tracks. Motors may engage in a catch‐like state, enabling cargo translocation in concert with MT motion, which is a process distinct from traditional motor stepping. In this hypothesis essay, we survey the current understanding of directional cargo transport and introduce a model in which cargoes co‐migrate with moving MTs. We also highlight how MT movement contributes to the spatiotemporal regulation of nuclear movements during neuronal migration in the developing brain.

### 1.1 Motor‐Stepping‐Based Directional Control on Fixed MTs

MT‐based transport has mostly been studied in vitro using MTs polymerized from purified tubulin proteins and immobilized on glass substrates for microscopy. Intracellular cargo trafficking has also been analyzed mainly using cells with predominantly stable MTs. Under these conditions, cargo transport is dictated by activities and interactions of molecular motors and cargoes, independent of MT movement. There are many review articles on this topic, which provide a comprehensive overview of MT‐based transport mechanisms [1, 9]. Here, we briefly discuss several key factors that influence directional control through motor regulation with examples from recent studies (Figure 1B):

1. *Stochastic binding*: Motors dynamically bind to or dissociate with adaptors/cargo and MTs in a probabilistic manner, which affects transport efficiency and direction. Recent evidence from live‐cell imaging supports the notion that the initiation of endosome transport toward MT‐minus‐ends is regulated by the stochastic assembly of dynein motor complexes and the kinetics of motor‐cargo interactions [10].
2. *Adaptor regulation*: Adaptor proteins on cargoes specify the motors that are recruited, thereby governing transport directionality. Adaptor molecules have been shown to regulate the activities of motors as well as coordination between opposing motors [11, 12]. For instance, intracellular transport of mitochondria is regulated by two adaptor molecules, TRAK1 and TRAK2, with TRAK1 preferentially binding kinesin‐1 and TRAK2 binding both kinesin‐1 and dynein [13]. Synergistic TRAK1 and TRAK2 activities are essential for proper distribution of mitochondria in neuronal dendrites [13, 14].
3. *Cargo properties*: The physical properties of cargoes, including their shape, size, and softness, influence recruitment and arrangement of motors on their surfaces, the generation of resistance force against the crowded cytoplasm, and the generation of complex cargo behaviors such as rotation and compression [15, 16]. Studies have shown that dynein motors generate greater collective forces than kinesin, suggesting larger cargoes preferentially undergo dynein‐mediated transport, with cargo size and motor density influencing transport dynamics [17, 18].

### 1.2 Directional Transport Influenced by the Local Intracellular Environment

**FIGURE 2:**
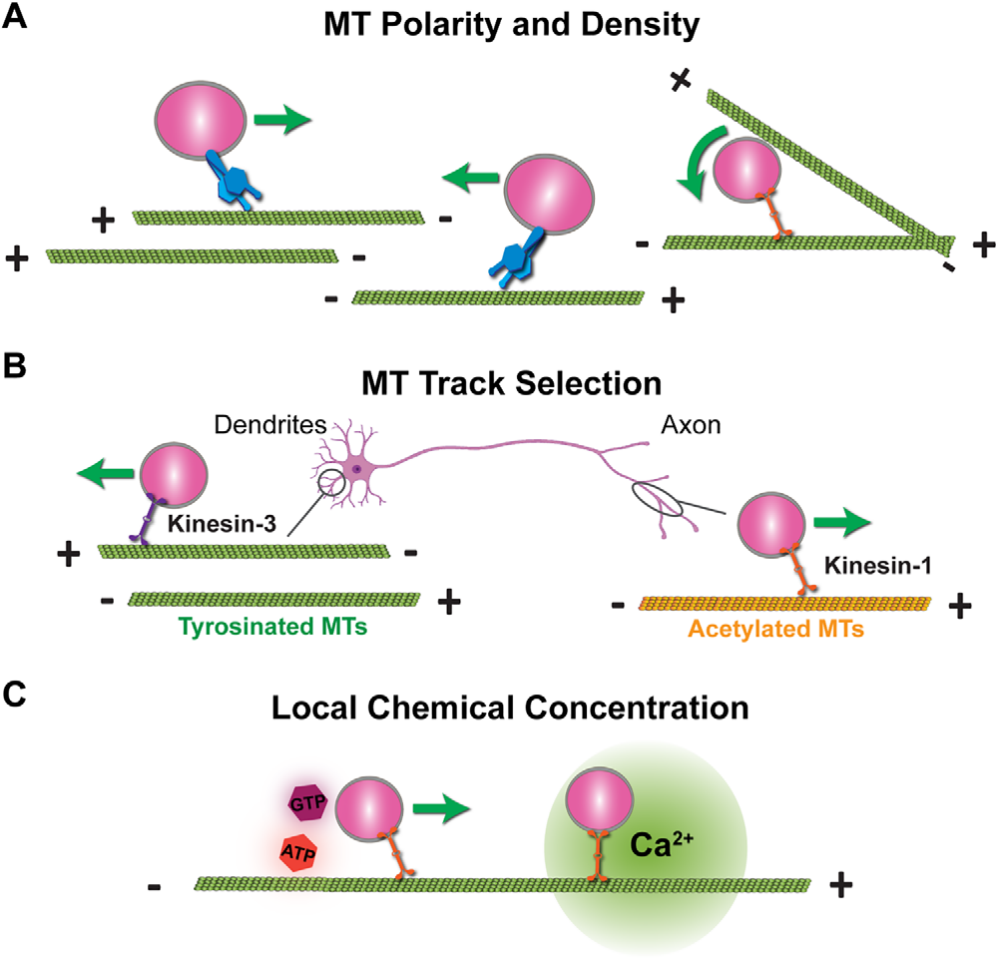
*Directional cargo transport influenced by local intracellular environment*. (A) Cargoes undergo directional transport or rotation depending on the organization of local MT tracks and bidirectional motors. (B) In the dendritic and axonal compartments of neurons, MTs with different PTMs recruit different types of kinesin motors, resulting in different patterns of cargo transport. (C) Cargo transport can be regulated by intracellular ATP, GTP, and calcium (Ca^2+^).

In living cells, the intracellular environment imposes additional complexities on cargo transport. Beyond motor activities, the following factors also contribute to directional control:

1. *MT arrangement*: cargo transport pathways are influenced by the arrangement of polarized MTs (Figure 2A) [19, 20]. The local density of MTs also affects the frequency of transport initiation [21, 22, 23, 24].
2. *MT track selection*: Dynein and kinesin motors selectively utilize specific MT subsets based on local post‐translational modifications (PTMs) and structural properties of MTs [25, 26, 27]. For instance, in neurons, Kinesin‐1 selectively binds to acetylated MTs to transport cargo to the axon, while Kinesin‐3 binds to tyrosinated MTs to deliver cargo to both axons and dendrites (Figure 2B) [28, 29]. MT‐binding proteins like tau and MAP7 also affect transport through interactions with motors or adaptors [30, 31]. Mechanical stress to MTs also affects motor‐driven transport [32, 33].
3. *Local chemical concentration*: In some cases, motor proteins or their adaptors sense the local chemical environment in specific subcellular compartments (Figure 2C). In vitro studies have demonstrated that ATP availability affects motor stall force and velocity [34]. GTP also plays an important role, where GTP hydrolysis by Rab GTPases regulates the interaction between specific effector‐motor complexes and cargo organelles. For example, GTP‐bound Rab7 recruits RILP‐dynein for retrograde transport of late endosomes/lysosomes. Additionally, Rab10 recruits JIP1‐kinesin‐1 for anterograde transport of post‐Golgi vesicles, and ARF6 recruits JIP4 to control motor switching between kinesin‐1 and dynactin for bidirectional endosome trafficking [35, 36, 37, 38]. TRAK1/2 delivers mitochondria at sites of elevated Ca^2+^ concentration in neurons by changing the composition of the TRAK‐motor complex when Ca^2+^ binds to the TRAK‐binding protein Miro1 [39, 40, 41, 42].

### 1.3 Lessons From Neuronal Nuclear Translocation in Migrating Neurons

On top of the mechanisms discussed above, we hypothesize that, in cells where MTs undergo directed movements, cargoes can also attach and hitchhike onto MTs via adaptors and MT motors. Here, we highlight the findings from our recent publication focusing on the transport mechanism for the nucleus in migrating neurons, which reaches the conclusion that organelles co‐migrate with advancing MTs [43].

**FIGURE 3:**
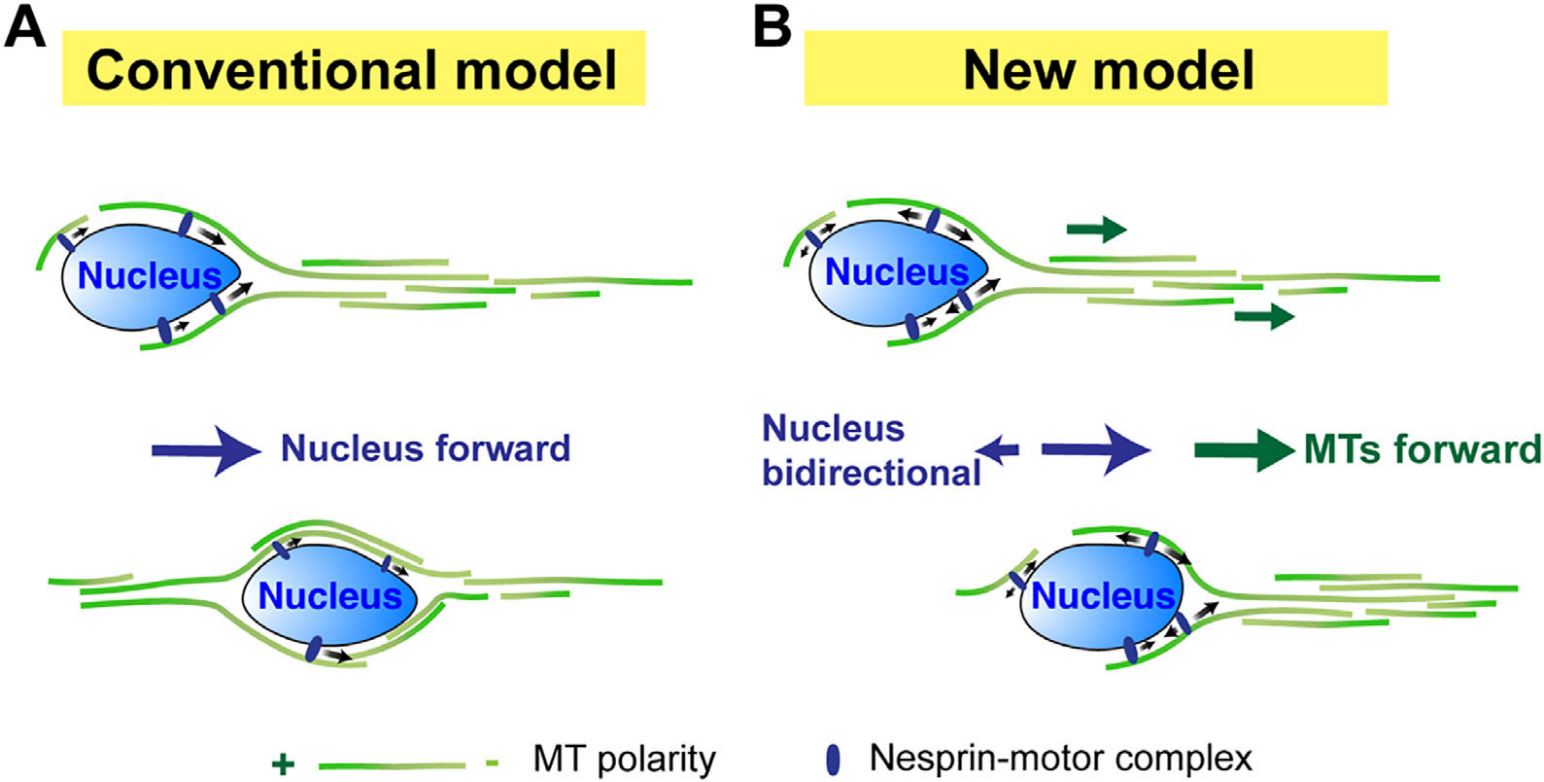
*Lessons from neuronal nuclear translocation in migrating neurons*. (A) The conventional model is that the nucleus is transported forward by dynein motors along stationary MTs. (B) The new model is that the nucleus undergoes bidirectional movements driven by the coordination of dynein and kinesin‐1 motors, while the peri‐nuclear MT tracks move forward, bringing the nucleus forward.

Translocation of the nucleus is critical for the proper positioning of neurons during brain development. Unlike the conventional view that dynein is the major player regulating the forward nuclear translocation (Figure 3A), we showed that dynein and kinesin‐1 are co‐dependent and closely coordinated via a nucleo‐cytoskeletal adaptor Nesprin‐2. We further demonstrated that Nesprin‐2 mediates bidirectional movements of cargoes along MTs, with only a slight bias toward minus ends (60% minus‐end‐directed and 40% plus‐end‐directed), which, statistically, could not efficiently drive forward nuclear translocation. Consistent with previous studies [44], we observed forward movements of peri‐nuclear MTs that were tightly coupled with nuclear forward steps in migrating neurons. Based on these results, we proposed that the nucleus can advance via coupling to moving MT tracks, rather than being transported on stationary MTs (Figure 3B). The previously characterized saltatory leaping motion of the neuronal nucleus might therefore be associated with the timing of MT forward sliding [45].

Beyond nuclear movements during neuronal development, we posit that the proposed model of MT‐movement‐aided cargo transport is applicable to diverse cargoes and systems, provided that key criteria, including cargo‐MT tethering and directional MT movement, are met. Emerging evidence suggests that dynamic MT movement can directly drive cargo transport, particularly in cells undergoing drastic morphological changes [46, 47, 48]. Beyond developmental contexts, MT movement also operates in mature neurons to maintain axonal MT polarity, to regulate growth and pruning of dendritic spines, and to prime neurite regeneration upon injury [49, 50, 51, 52]. Moreover, as reviewed by Lu and Gelfand [53], MTs exhibit sliding motion in diverse cell types outside neurons, including epithelial cells, fibroblasts, and Drosophila S2 cells, further indicating that MT‐movement‐aided transport is likely to operate broadly. We would like to stress that this hypothesized mechanism is complementary to the motor‐stepping‐dependent transport. Take multinucleated myotubes as examples, the distribution of nuclei is driven not only by motor‐dependent transport but also by pulling or sliding movements of MTs emanating from nuclei [54, 55, 56].

### 1.4 Hypothesized Mechanisms for Cargo‐Track Tethering

**FIGURE 4:**
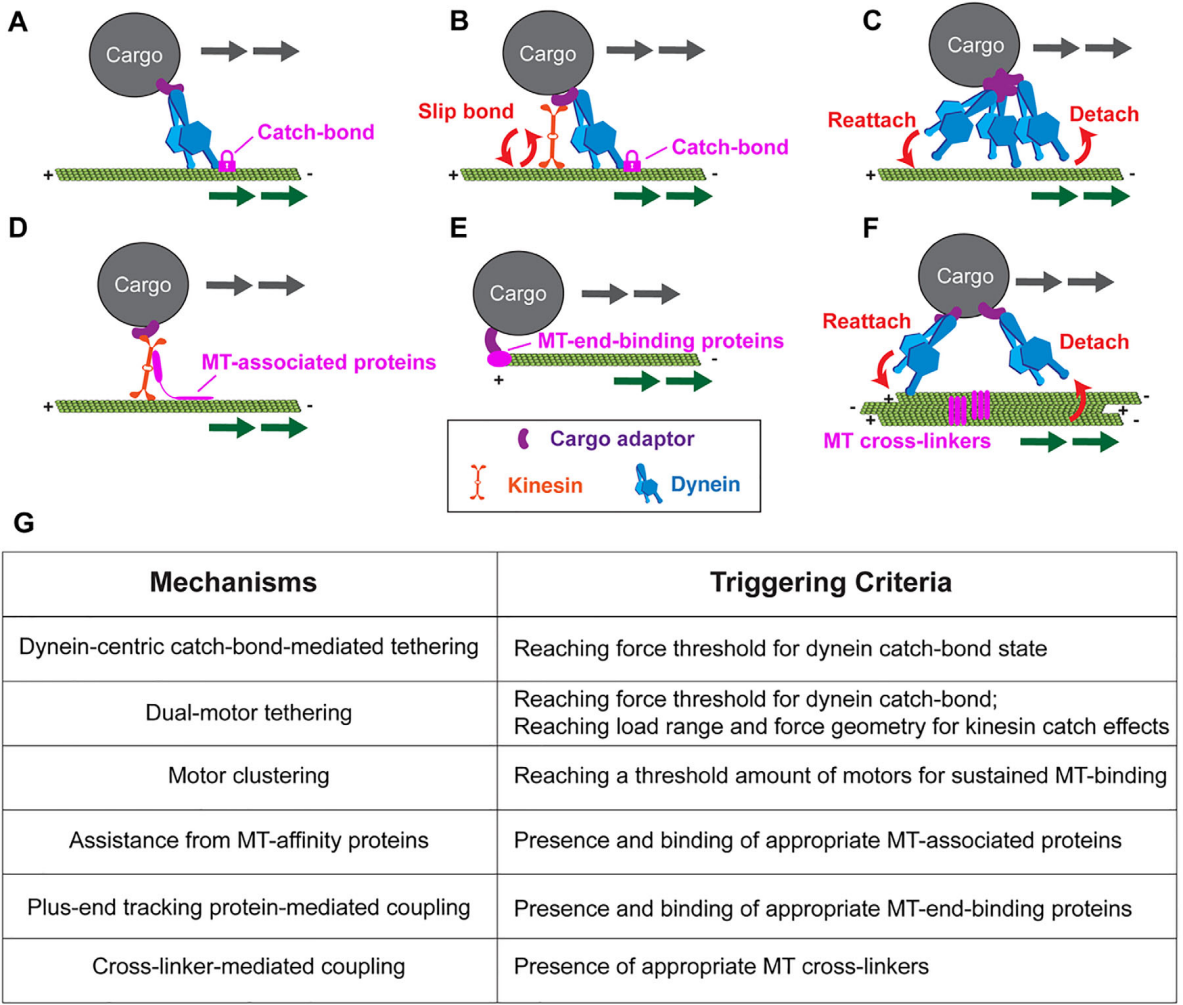
*Hypothesized mechanisms for cargo‐MT tethering during co‐migration*. (A) Under opposing load, dynein enters a catch‐bond state that stabilizes microtubule binding and prolongs attachment. (B) Kinesin‐1 exhibits slip‐bond behavior while dynein provides catch‐bond anchoring. Their complementary properties may sustain cargo‐MT coupling. (C) High local motor density enables statistical rebinding, maintaining contact through avidity effects. (D) MT‐associated proteins enhance tethering through multivalent interactions that recruit motors and potentiate motor‐MT interactions. (E) End‐binding proteins facilitate cargo docking onto dynamic MT plus ends. (F) MT bundling creates multivalent rails that distribute force among motors and facilitate lateral switching between adjacent filaments. (G) Summary of proposed tethering mechanisms and their triggering criteria. Arrows indicate MT movement (green) and cargo transport direction (gray).

A key question arising from the cargo‐MT co‐migration hypothesis is how cargoes are anchored onto moving MTs. When large cargoes with high inertia and viscous drag experience relative motion with MTs, tensile or compressive loads are generated on cargo‐bound motors or adaptors. Strong cargo‐MT coupling is required to prevent motor detachment and enable co‐migration. We propose six complementary mechanisms for load‐dependent tethering (Figure 4):

1. *Dynein‐centric catch‐bond‐mediated tethering*: We posit that dynein motors can enter a catch‐bond state, locking the connection between cargo and MTs, which counteracts the resistance of cargo movements (Figure 4A). Catch‐bond behaviors, first described in cell adhesion molecules including FimH, integrin, and selectin, involve force‐induced structural stabilization that prolongs bond lifetime under mechanical stress [57, 58, 59, 60]. For MT motors, dynein exhibits robust catch‐bond behavior under an opposing load, particularly when multiple motors work as a team on large cargoes, leading to prolonged stalling, higher collective forces, and reduced detachment [17, 61]. Mechanistically, tension transmitted from dynein's cargo‐attached tail to the microtubule‐binding domain (MTBD) stabilizes high‐affinity MTBD conformations, slowing detachment, and extending contact lifetime. Accessory factors, including dynactin and LIS1, may further stabilize this load‐bearing state [5, 62]. A dynein‐mediated catch‐bond has also been predicted to accelerate cargo transport, allowing more efficient delivery [63]. While a theoretical study suggests that dynein exhibits dynamic catch‐bonding, which is dependent on ATP hydrolysis [64], another study of Myosin I suggests load‐dependent ADP trapping maintains high‐affinity binding [65].
2. *Dual‐motor tethering*: While dynein provides robust catch‐bond tethering, kinesin predominantly exhibits slip‐bond behavior with shorter lifetimes under high opposing load, characterized by rapid rebinding, load‐direction sensitivity, and slip‐catch‐slip transitions (Figure 4B) [61]. Load direction significantly affects kinesin force generation [66, 67], suggesting mechanosensitive conformational plasticity in kinesin's MTBD, although the evidence for kinesin's catch‐bond is less well established than for dynein. In consort, however, load‐dependent reattachment kinetics of kinesin‐1 or kinesin‐2 may aid cargo tethering together with dynein [7, 68, 69, 70, 71].
3. *Motor clustering*: High local motor density increases effective on‐rates and avidity, extending tether lifetimes through statistical rebinding (Figure 4C). Teams of motor molecules have been shown to exhibit anisotropy in detachment rates for producing greater force to transport large cargoes [72, 73]. Under opposing force, one motor momentarily detaches, but another motor of close proximity immediately engages, keeping the cargo connected to MTs. Dynein clustering into lipid microdomains on the phagosome has been reported to support MT‐based transport [74], which may also function in MT tethering. This mechanism shows weaker load dependence but stronger copy‐number dependence than catch‐bonds.
4. *Assistance by MT‐affinity proteins*: Another possibility is that MT‐binding proteins enhance tethering by coupling motors and MTs through multivalent interactions (Figure 4D). For instance, MAP7, despite relatively weak direct lattice affinity, recruits and potentiates kinesin‐1, increasing landing rates and enhancing cargo tethering [75, 76, 77]. Other MAPs may contribute through avidity effects when multimerized or clustered around cargo attachment sites.
5. *Plus‐end tracking protein‐mediated coupling*: Extending from the MT‐binding protein model, another possibility involves the leveraging of MT end‐binding proteins (EBs), which bind to GTP‐tubulin caps, for end‐tracking tethering onto dynamic MTs (Figure 4E). MT end‐associated proteins, such as cytoplasmic linker protein 170 (CLIP‐170), EB‐1‐interacting adenomatous polyposis coli (APC), and EB‐1‐interacting stromal interaction molecule 1 (STIM1), facilitate docking and plus‐end‐tracking of motors or cargoes [78, 79, 80, 81, 82]. Phase separation mechanism may also contribute to cargo tethering at MT plus‐ends [83]. In this scenario, co‐migration correlates with MT growth rate and plus tip occupancy, with lifetimes decreasing when growth diminishes, independent of motor load signatures.
6. *Cross‐linker‐mediated coupling*: MT bundling may also strengthen cargo‐MT tethering by creating closely spaced, multivalent rails that boost effective affinity, rebinding, and load tolerance (Figure 4F). When MTs are bridged by cross‐linkers like PRC1 or MAP65, cargoes with multiple motors or weak MT‐binding modules maintain contact across adjacent filaments through spatial redundancy and geometric confinement [84, 85, 86]. Bundles enable force distribution among engaged motors and lateral “rail switching” without 3D escape. Intriguingly, the association time of kinesin‐1 motors is increased on MT bundles with mixed polarities and proper spacing, but not on parallel MT bundles [86]. Thus, this model might be more pronounced in antiparallel bundles like the midzone of mitotic spindles than in neuronal parallel bundles [28, 87]. Thus, with cross‐linker‐mediated coupling, cargo co‐migration strongly depends on the presence of cross‐linkers and the MT geometry.

There may be potential synergistic or antagonistic cross‐talk between these mechanisms. Future investigation is needed to catalog all of the interacting molecules and reveal the quantitative criteria for activating/deactivating tethering, including load range, cargo geometry, motor copy numbers, and timescales for interactions.

### 1.5 Hypothesized Mechanisms for Directed MT Movements

**FIGURE 5:**
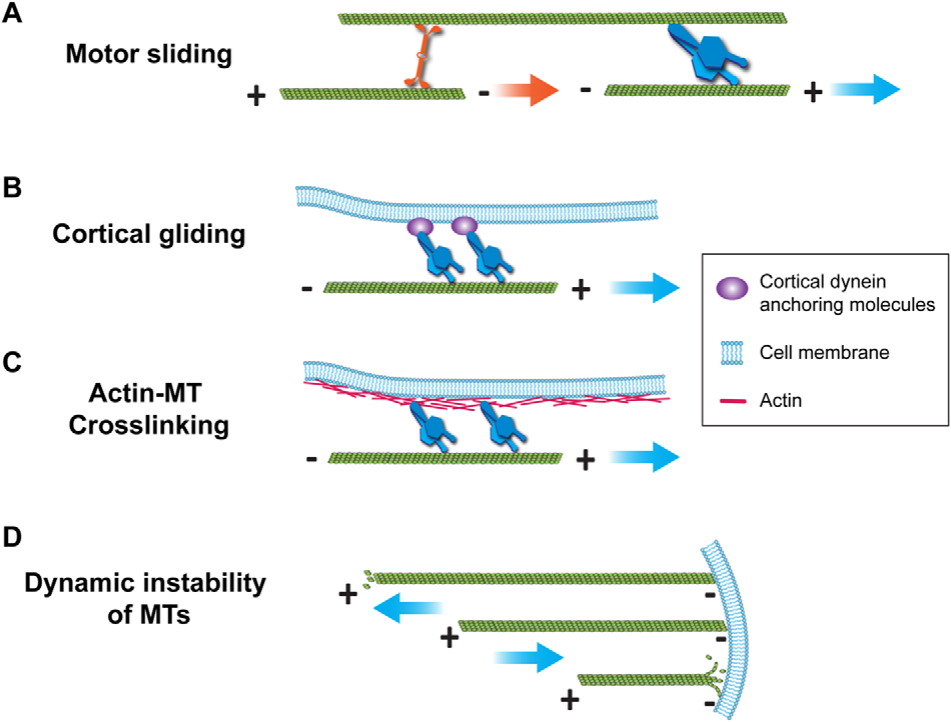
*Potential mechanisms for MT movements*. (A) When attached to a less movable MT track, kinesin/dynein motors can slide the shorter MT filaments against the stable MT. (B) When anchored onto the cell cortex through linking molecules, dynein can slide MTs against the cell membrane. (C) When cross‐linked with cortical actin, dynein can also slide MTs against the cell cortex. (D) Polymerization or depolymerization of MTs against the cell membrane generates MT movements. Arrows indicate the directional movements of MTs.

For cargo to be translocated by MT motion, directional MT movement must be actively generated and regulated. We hypothesize the following potential mechanisms based on available literature (Figure 5):

1. *Motor sliding*: MTs can be transported along other MTs via motor proteins [47, 88, 89]. In the presence of long and stable MTs acting as a stationary platform, smaller MT filaments can move along the stable MTs by motor‐driven sliding (Figure 5A). It has been reported that kinesin‐1 generates MT sliding in both Drosophila and mammalian cells [90, 91, 92, 93]. Aside from sliding motors, kinesin‐5, kinesin‐6, and kinesin‐12 can act as brakes on MT movements by binding and locking two MTs simultaneously [49, 94, 95, 96]. Deletion of kinesin‐5 in growing neurons results in excessive MT penetration into the axon growth cone, indicating that it functions as a brake to counteract MT sliding [97]. A localized dynein‐dynactin complex can also generate MT sliding, resulting in contractile stresses within the cytoskeleton [98].
2. *Cortical gliding*: There is evidence that dynein anchored to the cell cortex via membrane‐bound molecules, such as nuclear mitotic apparatus protein (NUMA) and scaffold protein LGN, can generate MT movement against the cell membrane [99]. Cortical dyneins drive directional MT movement by sliding the MTs toward their plus‐ends (Figure 5B) [100]. Dynein‐dependent MT gliding against the cell cortex has been observed in Drosophila neurons [49] and in ovaries [101]. It has also been reported that a dynein and dynactin complex stabilizes dynamic MTs, prevents depolymerization, and facilitates MT sliding [102, 103].
3. *Actin‐MT crosslinkers*: Spectrin or other crosslinkers may also contribute to MT movements. The actin scaffold underneath the cell membrane can also interact with MTs via actin‐MT crosslinker molecules such as Drebrin (Figure 5C) [44]. Actin contraction may, therefore, transduce forces to MTs and generate MT movements.
4. *Dynamic Instability of MTs*: Polymerization and depolymerization of MTs that are anchored to stable structures, such as the cell membrane, can exert pushing or pulling forces for cargo co‐transport (Figure 5D). Such dynamic instability of MTs has been shown to generate forces during chromosome segregation in mitosis and nuclear positioning in budding yeast [104, 105]. Gros et al. [106] have shown that centrosome transport in functioning T cells relies on both MT‐capture‐shrinkage and MT‐sliding, indicating that various mechanisms for generating MT movements are not mutually exclusive.

Experimental evidence is needed to differentiate active MT movements from cytoplasmic flow. Although bulk cytoplasmic flow may also drive directional organelle movements [107, 108, 109], it is more continuous and uniform in both time and space, but less specific to individual cargoes. Bulk transport by cytoplasmic flow is associated with large cells with diameters over 100 µm, which may not be applicable for smaller cell compartments like neuronal processes [110]. Based on our observation in neurons, the nucleus co‐transported with MTs exhibits a pause‐and‐run movement, with spatiotemporally regulated MT and cargo dynamics, but more work is needed to understand how the timing and mechanics between cargo tethering activation are coordinated with directional MT movements [43].

### 1.6 Physiological Relevance and Disease Implications

Defects in cargo transport, particularly in neurons, are linked to various neurodevelopmental and neurodegenerative diseases. For instance, lysosome maturation relies on directed endo‐lysosome transport in neurons, and mutations in related transport proteins have been associated with disorders such as Amyotrophic Lateral Sclerosis (ALS) and Alzheimer's disease [111, 112, 113]. Additionally, MT movement plays a role in neuronal polarity establishment, including axon development and maturation [114, 115, 116]. Furthermore, MT‐based cargo transport and dynamic MT movements also play an important role in axon regeneration after nerve damage [52]. Understanding the contribution of MT mobility to the regulation of transport will provide insights into disease pathology and potential therapeutic targets.

## 2 Future Investigation

To investigate the hypothesis that cargos are coupled with moving MTs to achieve directed transport, biophysical modeling incorporating in vitro motor transport parameters and intracellular MT movements is required to predict the behaviors of intracellular cargo transport more accurately. Stoke's law has been used to estimate forces required for cargoes of varying sizes to move at certain velocities, which may be utilized to calculate the required force for motors to enter catch‐bond states depending on the cargo size and the number of motors for cargo tethering [117, 118]. Computational models for simulating motor‐driven MT movements are available [119], which can be combined with motor‐driven cargo dynamics to predict transport outcomes. In addition, advanced imaging techniques, such as super‐resolution live‐cell imaging and expansion microscopy, together with improved photoconvertible probes, are needed to capture cargo and MT dynamics with higher spatiotemporal resolution, providing direct evidence for the postulated underlying mechanisms. Simultaneous imaging of different intracellular organelles will help to assess whether co‐movements of organelles are specific to particular cargoes. Last but not least, identifying key proteins supporting cargo tethering or driving MT movements will facilitate in vitro reconstitution for better recapitulation of intracellular events under physiological and disease conditions.

## Author Contributions

Conceptualization: **C.Z**., **M.K**. Writing – original draft: **C.Z**. Writing review – & editing: **C.Z**., **M.K. **.

## Conflicts of Interest

The authors declare no conflicts of interest.

## Data Availability

Data sharing not applicable to this article as no datasets were generated or analyzed during the current study.
